# Sensory nerves mediate spontaneous behaviors in addition to inflammation in a murine model of psoriasis

**DOI:** 10.1096/fj.201800395RR

**Published:** 2018-09-11

**Authors:** Xenia Kodji, Kate L. Arkless, Zizheng Kee, Simon J. Cleary, Aisah A. Aubdool, Elizabeth Evans, Paul Caton, Simon C. Pitchford, Susan D. Brain

**Affiliations:** * British Heart Foundation (BHF) Cardiovascular Centre of Research Excellence, Vascular Biology and Inflammation Section, King’s College London, London, United Kingdom;; † Sackler Institute of Pulmonary Pharmacology, Institute of Pharmaceutical Science, King’s College London, London, United Kingdom;; ‡ Diabetes Research Group, Division of Diabetes and Nutritional Sciences, King’s College London, London, United Kingdom

**Keywords:** TRP channels, neuropeptides, macrophages, oxidative stress, peroxynitrite

## Abstract

Psoriasis is characterized by keratinocyte hyperproliferation, erythema, as well as a form of pruritus, involving cutaneous discomfort. There is evidence from both clinical and murine models of psoriasis that chemical or surgical depletion of small-diameter sensory nerves/nociceptors benefits the condition, but the mechanisms are unclear. Hence, we aimed to understand the involvement of sensory nerve mediators with a murine model of psoriasis and associated spontaneous behaviors, indicative of cutaneous discomfort. We have established an Aldara model of psoriasis in mice and chemically depleted the small-diameter nociceptors in a selective manner. The spontaneous behaviors, in addition to the erythema and skin pathology, were markedly improved. Attenuated inflammation was associated with reduced dermal macrophage influx and production of reactive oxygen/nitrogen species (peroxynitrite and protein nitrosylation). Subsequently, this directly influenced observed behavioral responses. However, the blockade of common sensory neurogenic mechanisms for transient receptor potential (TRP)V1, TRPA1, and neuropeptides (substance P and calcitonin gene-related peptide) using genetic and pharmacological approaches inhibited the behaviors but not the inflammation. Thus, a critical role of the established sensory TRP–neuropeptide pathway in influencing cutaneous discomfort is revealed, indicating the therapeutic potential of agents that block that pathway. The ongoing inflammation is mediated by a distinct sensory pathway involving macrophage activation.—Kodji, X., Arkless, K. L., Kee, Z., Cleary, S. J., Aubdool, A. A., Evans, E., Caton, P., Pitchford, S. C., Brain, S. D. Sensory nerves mediate spontaneous behaviors in addition to inflammation in a murine model of psoriasis.

Psoriasis is a common skin inflammatory condition that affects 2% of the population globally, characterized by skin scaling, erythema, and an inflammatory cell-rich cutaneous microenvironment (1). Psoriasis has long been associated with sensory discomfort, such as cutaneous pain and chronic itch (2, 3). There is an increasing availability of antipsoriatic treatments, but they often have adverse effects, high costs, and differing effects on inflammation and pruritic components (4, 5). Hence, psoriasis remains an unmet clinical need, and novel therapies with better efficacy need to be developed targeting both skin pathology and, specifically, the discomfort. To study the integrative mechanisms in psoriasis, *in vivo* murine models have proven to be essential, such as the Aldara mouse model. Aldara cream (Meda, Solna, Sweden) is an immunomodulatory cream, which contains 5% imiquimod, a TLR 7/8 agonist. Consecutive, repeated application of Aldara on the mouse dorsal skin results in a psoriasis-like phenotype (6). Additionally, this model possesses a scratching phenotype (7) and was shown to have the greatest similarity to human psoriasis when using mice with a C57/BL6J background (8), thus providing an important tool for studying the mechanisms involved in human psoriasis.

One of the proposed therapeutic approaches being targeted for psoriasis is the cutaneous sensory nerves (nociceptors). Those nerves comprise C and Aδ fibers that release neuropeptides; of which substance P (SP) and calcitonin gene-related peptide (CGRP) are best characterized upon activation by various mechanisms that include transient receptor potential (TRP) channels [*e.g.*, TRP ankyrin 1 (TRPA1) and TRP vanilloid 1 (TRPV1)] (9). The activation of TRP channels mediates neurogenic inflammation in skin as well as contribute to pain in arthritis-associated conditions (9, 10). Surgical- and chemical-mediated sensory denervation targeting those nociceptors improves psoriasis in humans as well as in murine models (11, 12), but the mechanisms are unclear. Additionally, those small-diameter nociceptors are important in itch, as well as pain, sensations (13, 14). The roles of the cutaneous sensory nerves have recently been highlighted in various murine models of skin inflammatory conditions, such as atopic dermatitis and pruritus (15, 16). Sensory mediators, such as TRPA1 and neuropeptides, have significant roles in mediating both the underlying inflammation as well as the nocifensive behaviors, such as pruritus (15, 16), highlighting a potential therapeutic use in targeting the sensory nerves in skin inflammatory diseases. Indeed, topical capsaicin cream, which, when repeatedly applied, results in sensory denervation (17), and has been consistently shown to be beneficial in minimizing both psoriasis-associated skin lesions and cutaneous discomfort, once the initial burning has subsided (18). In contrast to atopic dermatitis, the role for sensory nerves and associated mediators in psoriasis requires further investigation, especially because antipsoriatic treatments show inconsistent efficacy in psoriasis-associated pruritus (2, 5). Indeed, a study (19) showed that chemical sensory denervation with resiniferatoxin markedly improved psoriasis and proposed an early involvement of sensory nerves in regulating dermal dendritic cell activation in psoriasis in the murine Aldara model. However, that study focused on the early regulation of dermal dendritic cell activation by sensory nerves during the development of psoriasis; it did not further examine the underlying inflammatory and pruritogenic mechanisms involved. We have recently shown (20) that TRPA1 acts in a protective manner in the murine Aldara model of psoriasis using TRPA1-deleted mice or when a TRPA1 antagonist was administered repeatedly; we did not, however, study the effect of sensory nerve deletion or blockade. Here, we investigated the TRPV1/TRPA1 sensory pathways and neuropeptides, mainly SP and CGRP, through use of resiniferatoxin depletion (21, 22). We show that sensory nerves contribute to macrophage-induced inflammation, which is associated with reactive oxygen species/reactive nitrogen species (ROS/RNS) generation, forming an integral pathway to the ensuing inflammation and sensory discomfort. Moreover, we reveal that, although the established TRP/neuropeptide pathways do not influence the ongoing inflammation, they benefit the observed cutaneous discomfort, providing a potential therapeutic target.

## MATERIALS AND METHODS

### Animals

All animal procedures were carried out according to the United Kingdom’s Home Office Animals (Scientific Procedures) Act 1986 and the Animal Research: Reporting of *In Vivo* Experiments guidelines. This study was approved by King’s College Animal Care and Ethics Committee. Male, 6–8-wk (20–25 g) C57/BL6J mice (Charles River Laboratories, Wilmington, MA, USA) were used in this study. Various genetically modified mice [TRPV1 knockout (KO) and α-CGRP KO] were raised on a C57/BL6J background and were a kind gift to Prof. S. D. Brain (TRPV1 KO; Merck, Kenilworth, NJ, USA) and by Dr. Anne-Marie Salmon (α-CGRP KO; Institut Pasteur, Paris, France) (23). Transgenic animals were maintained and bred in-house in a climatically controlled environment (22°C) and exposed to 12/12-h light/dark cycle. All recovery procedures were performed under 2% isoflurane (Isocare; Animalcare, York, United Kingdom) with 2% oxygen. All procedures were terminated by cervical dislocation. Throughout the *in vivo* and *ex vivo* experiments, the animals were randomized, and the investigators were blinded to the genotype/treatment groups.

### Induction of psoriasis-like skin inflammation and dorsal skin blood flow measurement

The mouse dorsal skin area was shaved and depilated (Veet; Reckitt Benckiser Health Care, Hull, United Kingdom) on the first day after the application of Aldara cream. Four square centimeters of dorsal skin area was treated with 75 mg of Aldara cream or Vaseline (petroleum jelly; Unilever, London, United Kingdom) for a consecutive 4 d, according to previously published work by Roller *et al.* (24). Samples were collected on d 5. Daily double-fold skin thickness was measured with a micrometer (Farnell, Leeds, United Kingdom) with 0.1-mm accuracy, and the change in thickness was calculated against d 0 thickness. For each day, the mean of 3 readings were taken (1 each at the top, middle, and lower areas of the dorsal skin). Cutaneous blood flow in the dorsal skin was measured using the full-field laser perfusion imager (Moor Instruments, Axminster, Devon, United Kingdom). The scanner was placed 20 cm above the anesthetized mice (placed on a homeothermic mat maintained at 36°C). The scanner then emitted a laser that penetrated to a 1-mm depth in the area of interest and was previously used to measure hind paw and ear blood flow in real time (22, 25). The settings used were as follows: high-resolution capture (25 frames, 1 s/frame), gain: auto, exposure: 20 ms. The blood flow was measured until a stable blood flow reading was obtained for 5 min continuously, and the mean blood flux units (blood flow) were calculated with the MoorFLPI Review 3.0 software (Moor Instruments) for the last 2 min of measurement. Regions of interest were highlighted around the treated area, which were kept consistent throughout the treatment period.

### Spontaneous behavior observations

Mice were acclimatized to the behavioral room for at least 2 h before observations, and baseline measurements were taken across 2 d before disease induction (d −2 and −3). The subsequent observations were then carried out in a see-through behavior chamber (20 × 20 × 14 cm; Ugo Basile, Gemonio, Italy). During treatment days, behaviors were observed daily at 4 h after skin treatment. A 30-min observation of nocifensive/pruritic behaviors was performed, noting: *1*) hind paw scratching: 1 event was defined as hind paw returning to the ground, *2*) biting/licking: 1 event was defined by touching of the mouth and nose to the treated dorsal area, and *3*) flinching: a rotating/rippling movement across the dorsal area (26). Hind paw scratching was shown to indicate pruritic sensations (27), whereas biting/licking events and flinching were indicative of cutaneous discomfort and nocifensive behaviors. These behaviors were previously associated with discomfort or even pain, similar to observations after the intrathecal administration of SP (28) or intraplantar formalin in rats (26). Indeed, clinical psoriasis has been associated with cutaneous discomfort and pain in addition to pruritus (3); hence, these behaviors were measured to indicate psoriasis-associated cutaneous discomfort in this model. Total d 4 spontaneous behaviors were calculated as the total number of biting/licking events and flinching responses.

### Pharmacological treatments regime

Sensory denervation was induced with a daily resiniferatoxin (RTX) injection for 4 d (0.3 mg/kg s.c. into the nape of the neck; MilliporeSigma, Burlington, MA, USA) or vehicle (10% Tween-80, 10% ethanol, 80% saline), as previously characterized by Aubdool *et al.* (22). Successful denervation was confirmed with a hot plate test, where mice were placed on a hot plate (55°C) for a maximum of 30 s. The time taken for mice to show discomfort (withdrawal latency), such as hind paw licking, biting, or flinching was recorded. Mice that did not show discomfort within the timeframe were removed from the hot plate, and the withdrawal latency was recorded as 30 s. For acute TRPA1 inhibition, vehicle (0.5% methylcellulose in water), HC030031 (100 mg/kg, i.p.; Bio-Techne, Minneapolis, MN, USA), or A967079 (60 mg/kg, oral; Alomone Labs, Jerusalem, Israel) was administered 30 min or 1 h, respectively, before spontaneous behavior measurements on d 4. For neuropeptide receptor antagonists and 4-hydroxy-2,2,6,6-tetramethylpiperidine-*N*-oxyl (tempol) treatments, once daily treatments were administered 30 min before topical skin treatment over 4 d. CGRP receptor antagonist BIBN4096 (3 mg/kg, i.p. daily; Bio-Techne) (29, 30) and/or NK_1_ receptor antagonist aprepitant (10 mg/kg, i.p. daily; AdooQ Bioscience, Irvine, CA, USA) (15, 31) or vehicle (5% DMSO in saline) were administered separately or in combination. Tempol (200 mg/kg, i.p.; Enzo Life Sciences, Farmingdale, NY, USA), a permeable superoxide dismutase (SOD) mimetic, or vehicle (saline) was also administered.

### Macrophage depletion study

To induce macrophage depletion *in vivo*, clodronate-containing liposomes (*http://clodronateliposomes.org*; Haarlem, The Netherlands), containing 5 mg of clodronate/ml of suspension, were used. Vehicle treatment consisted of PBS-containing liposomes. The injection dose was 10 ml of liposomes/kg of animals. Liposomes were administered intraperitoneally on d −3, 0, and 2 of Aldara treatment, as recommended by the suppliers, because it takes ∼3 d for complete depletion per the supplier’s instructions. That injection regime was chosen to ensure that thorough depletion was maintained throughout the study period.

### Histopathology

Frozen sections of dorsal skin tissues were embedded in the Optimal Cutting Temperature compound (Bright Cryo-M-Bed; Bright Instruments, Luton, United Kingdom) and were stored at −80°C for further processing. Next, 10 µm of frozen skin samples were sectioned with a cryostat (CM1520; Leica Microsystems, Wetzlar, Germany). Then, skin sections were stained with hematoxylin and eosin (H&E) following standard protocols. Sections were imaged and captured with the ProGres Microscope Camera (Jenoptik, Jena, Germany) with exposure settings of 1.5 ms, light at 7.0, and magnification at ×10. Epidermal thickness (µm) was quantified with ImageJ software (v.1.32; National Institutes of Health, Bethesda, MD, USA) and was measured between the epidermal surface and the epidermal–dermal junction, excluding the rete ridges. The average thickness was determined over an average of 6–10 sections/animal. For each skin section, we measured the whole length of the skin where possible (hence, at ×10 magnification, they were determined by ≥3–4 images). For each image, we took 8 points distributed evenly over the epidermis and averaged them. Hence, the data presented here is the value of average epidermal thickness/percentage of area coverage.

### Immunohistochemistry

Freshly sectioned skin samples were prepared and left to dry overnight before staining. Sections were fixed in 4% paraformaldehyde in PBS for 15 min, before rinsing under tap water and incubating in 3% H_2_O_2_ (Santa Cruz Biotech, Dallas, TX, USA) in ethanol for 10 min. The slides were then washed and immersed in PBS before blocking with 1% bovine serum albumin in PBS. All subsequent washing steps between incubations were performed in PBS. Primary antibodies (or blocking buffer for the negative control) used were rat anti-Lymphocyte Antigen 6 Complex Locus G6D (Anti-Ly6G, 1:10,000, BE0075-1; BioXCell, West Lebanon, NH, USA) or rabbit anti–ionized calcium-binding adapter molecule 1 (anti-Iba1; 1:750, 019–19741; Wako Pure Chemical Industries, Osaka, Japan) and incubated for 2 h. The sections were incubated in the appropriate secondary antibody for 1 h, followed by incubation in streptavidin–biotin horseradish peroxidase complex [VectaStain Elite ABC Kit (standard), 1:200 dilution of reagents A and B, following the protocol described; Vector Laboratories, Burlingame, CA, USA)] for 1 h. Slides were developed in 0.25 mg/ml 3,3′-diaminobenzidine (DAB) for 10 min in developing buffer containing 0.1 M Tris (pH 7.6) and 0.03% H_2_O_2_, resulting in dark-brown staining for cells expressing the relevant markers. Gill’s No. 2 hematoxylin solution (MilliporeSigma) was used to counterstain the sections, and sections were differentiated in 1% acid alcohol solution (MilliporeSigma) before slides were dehydrated in increasing alcohol concentrations and xylene. Coverslips were mounted and left to dry overnight.

For the percentage of area covered by DAB^+^ immune cells, automated quantification was performed by cropping the images around the dermal regions (where the inflammatory cell influx occurred), excluding the epidermal and subcutaneous fat layers to minimize nonspecific staining. Automated quantification was normalized to the size of the dermal region area being analyzed. Sections with excessive hair follicles may interfere with that process because of the dark-colored melanin pigments in C57/BL6J mice; hence, those sections were excluded from further analysis. The RGB images underwent a color-deconvolution step (32), which allowed the separation of DAB-specific colors from the hematoxylin counterstain. According to the strength of the DAB staining after color deconvolution, the threshold for staining was set at 0–150 (for Ly6G^+^ staining) and 0–180 (for Iba1^+^ staining), so that the deconvoluted RGB images were transformed into black and white images). This allowed for the quantification of DAB^+^ stain and minimized the detection of nonspecific background staining during particle analysis on ImageJ software.

### Western blotting

Dorsal skin samples for Western blotting were homogenized in SDS lysis buffer (50 mM Tris base, pH 6.8, 10% glycerol, 2% SDS) containing a protease-inhibitor cocktail (MilliporeSigma) and a phosphatase inhibitor (PhosStop; Hoffmann-La Roche, Basel, Switzerland). Skin samples were homogenized with TissueLyser II LT (Qiagen, Hilden, Germany), and the supernatants were collected and assayed. Then, 50 µg of protein/sample was prepared to load into a Mini-Protean Tetra Vertical Electrophoresis Chamber (Bio-Rad Laboratories, Hercules, CA, USA). The separated proteins were transferred and immobilized onto Immobilon-P polyvinylidene fluoride membranes (MilliporeSigma) at 20 V for 2 h. Nonspecific binding was blocked in 5% skimmed milk (or 1% bovine serum albumin for OxyBlot; MilliporeSigma) before the membrane was washed with PBS containing 0.1% Tween 20 between incubations. Membranes were incubated in primary antibody overnight at 4°C. Primary antibodies tested in this study were: *1*) mouse mAb anti–3-nitrotyrosine (anti–3-NT, 39B6, ab61392, 1:500; Abcam, Cambridge, United Kingdom), *2*) rabbit pAb anti–4-hydroxynonenal (anti–4-HNE; ab46545, 1:1000; Abcam), *3*) rabbit anti-dinitrophenylhydrazone (DNP) IgG antibody (Oxyblot Kit S7150, 90451, 1:150; MilliporeSigma), and *4*) mouse mAb anti–glyceraldehyde-3-phosphate dehydrogenase (GAPDH; AM4300, 1:5000; 37 kDa for loading control; Thermo Fisher Scientific, Waltham, MA, USA). Secondary antibodies used were: *1*) goat anti-rabbit IgG antibody (AP132P, 1:2000; MilliporeSigma), *2*) goat anti-mouse IgG antibody (AP124P, 1:2000; MilliporeSigma), and *3*) goat anti-rabbit IgG antibody (OxyBlot Kit, 1:300; MilliporeSigma). For detection, the membrane was incubated with 1 ml of ECL solution (Luminata Classico or Crescendo Western horseradish peroxidase substrate; MilliporeSigma) and was developed in the G-Box Gel Documentation System (Syngene International, Bangalore, India). Chemiluminescence images were captured with the Syngene 2D Gel imaging software, and densitometry analyses were performed with ImageJ software (the whole column was analyzed instead of specific bands at specific MW in keeping with the assessment of oxidative stress), with arbitrary intensity value expressed as a ratio to the corresponding values for the loading control (GAPDH).

### Quantitative RT-PCR

RNA extraction and purification from the dorsal root ganglia (DRGs) and the skin were performed with the RNeasy Microarray Tissue Mini Kit (Qiagen) following the manufacturer’s instructions. RNA content and quality were quantified with a Nanodrop 1000 spectrophotometer (Thermo Fisher Scientific) and were checked for RNA purity; 500 ng of the isolated RNA was prepared for reverse transcription into cDNA with the High-Capacity RNA-to-cDNA Kit (Thermo Fisher Scientific), as described in the manufacturer’s instructions. SensiFAST SyBR-Green No-Rox Kit (Bioline, London, United Kingdom) was used for real-time quantification of target gene amplification and quantitative PCR was performed on a Robotics CAS-1200 (Qiagen). Three reference genes were used [β-actin, HPRT1 (hypoxanthine phosphoribosyltransferase 1), β-2-microglobulin]. Using the GeNorm v.1.2 software (Biogazelle, Gent, Belgium), a normalization factor was calculated for each sample based on the geometric mean of the reference genes (33). Copies/µl of each gene were calculated by the Rotor Gene 6000 Series Software 1.7 (Qiagen). Primers sequences were as follows: β-actin, forward, 5′‑CACAGCTTCTTTGCAGCTCCTT‑3′, reverse, 5′‑TCAGGATACCTCTCTTGCTCT‑3′; α‑CGRP, forward, 5′‑AGCAGGAGGAAGAGCAGGA‑3′, reverse, 5′‑CAGATTCCCACACCGCTTAG‑3′; β2‑microglobulin, forward, 5′‑GTCGCTTCAGTCGTCAGCA‑3′, reverse, 5′‑TTGAGGGGTTTTCTGGATAGCA‑3′; C‑C motif chemokine ligand 5 (CCL5), forward, 5′‑TGCTCCAATCTTGCAGTCGT‑3′, reverse, 5′‑GCGTATACAGGGTCAGAATCAAG‑3′; HPRT, forward, 5′‑CCTGGTTCATCATCGCTAATC‑3′ reverse, 5′‑TCCTCCTCAGACCGCTTTT‑3′; IL‑1β, forward, 5′‑GGGCTGCTTCCAAACCTTTG‑3′, reverse, 5′‑TGATACTGCCTGCCTGAAGCTC‑3′; IL‑6, forward, 5′‑GGTGACAACCACGGCCTTCCC‑3′, reverse, 5′‑ACAGGTCTGTTGGGAGTGGTATCC‑3′; IL‑8, forward, 5′‑GGCATCTTCGTCCGTCCCT‑3′, reverse, 5′‑CCAACAGTAGCCTTCACCCA‑3′; IL‑17A: Mm_Il‑17a_1_SG Quantitect Primer Assay (200; QT00103278F; Qiagen); IL‑23p19, forward, 5′‑AATGTGCCCCGTATCCAGTG‑3′, reverse, 5′‑GCAGGCTCCCCTTGAAGAT‑3′; SP, forward, 5′‑AAGCCTCAGCAGTTCTTTGG‑3′, reverse, 5′‑TCTGGCCATGTCCATAAAGA‑3′; TLR3, forward, 5′‑GATGATGCAGTCTTTCCAGAGGGA‑3′, reverse, 5′‑GACAAAAGTCCCCCAAAGGAGTA‑3′; TLR7, forward, 5′‑AAGAAAGATGTCCTTGGCTCCCTTC‑3′ reverse, 5′‑TTTGTCTCTTCCGTGTCCACAT‑3′; TNF‑α, forward, 5′‑CGGAGTCCGGGCAGGT‑3′, reverse, 5′‑GCTGGGTAGAGAATGGATGAACA‑3′; TRPA1, forward, 5′‑AGGTGATTTTTAAAACATTGCTGAG‑3′, reverse, 5′‑CTCGATAATTGATGTCTCCTAGCAT‑3′; TRPM8, forward, 5′‑TTGTATTCCGGCTCCACTCTTC‑3′, reverse, 5′‑AGTTCCTGCTGACGGTGAAAA‑3′; and TRPV1, forward, 5′‑CAACAAGAAGGGGCTTACACC‑3′, reverse, 5′‑TCTGGAGAATGTAGGCCAAGAC‑3′.

### Statistics

Results are expressed as means ± sd and were analyzed on Prism 5 software (GraphPad Software, La Jolla, CA, USA). For data sets at various time points, such as dorsal skin blood flow and spontaneous behavior observations, 2-way repeated-measures ANOVA with a Bonferroni’s *post hoc* test was performed. For other data sets, 2-way ANOVA with Bonferroni *post hoc* test was performed. A value of *P* < 0.05 was considered statistically significant.

## RESULTS

### RTX-mediated sensory denervation protects against Aldara-mediated skin pathology and cutaneous discomfort

**Figure 1:**
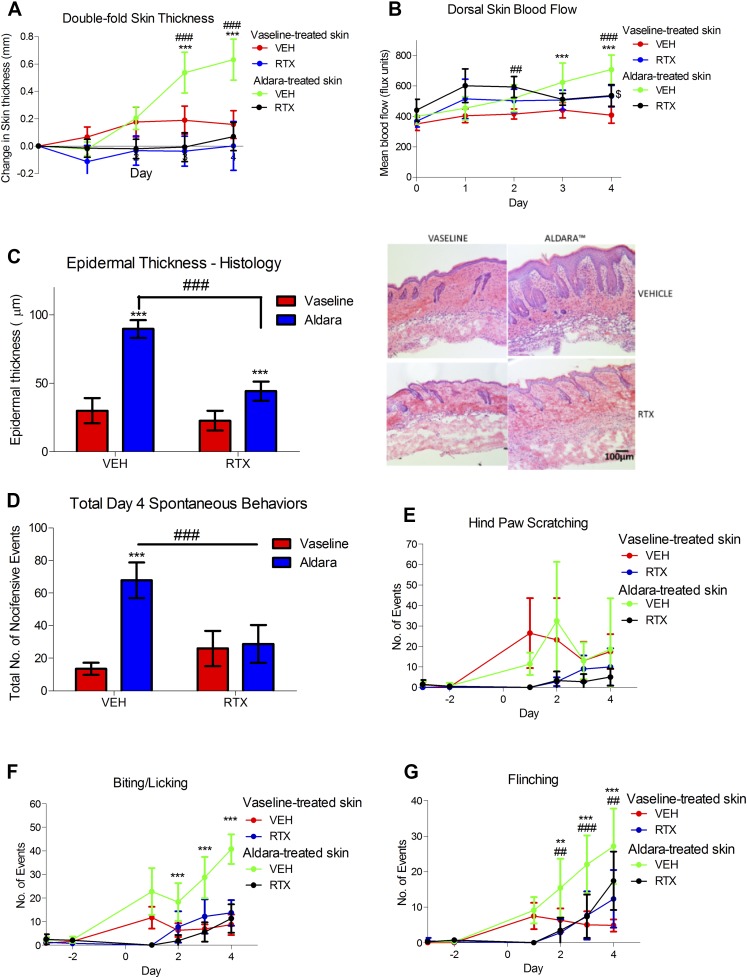
RTX-mediated sensory denervation protected against psoriasis-like skin pathology and associated discomfort. *A*, *B*) *In vivo* change in double-fold skin thickness (mm) (*A*) and dorsal skin blood flow (*B*). *C*) Epidermal thickness measured and representative images of H&E-stained skin samples sectioned at 10 μm thickness (right). Original magnification, ×10. Scale bar, 100 μm. *D*) Total spontaneous behaviors on d 4. *E*) Total number of hind paw scratching events. *F*) Total number of biting/licking events. *G*) Total number of flinching events. VEH, vehicle. Graphs represent means ± sd, and data were analyzed by repeated measures 2-way ANOVA with Bonferroni’s *post hoc* test; *n* = 5–7 animals/group.*P < 0.05, **P < 0.01, ***P < 0.001 between Vaseline *vs.* Aldara group, ^###^*P* < 0.001 between VEH *vs.* RTX, ^$^*P* < 0.05 between Vaseline VEH *vs.* Vaseline RTX.

Repeated Aldara treatment to the mouse dorsal skin resulted in a consistent increase in double-fold skin thickness *in vivo*, partly from acanthosis (epidermal thickening) (**Fig. 1*A*, *C***), similar to clinical psoriasis. In addition, erythema is associated with lesional skin in psoriasis. To assess that, we used the laser speckle technique to quantitatively measure mouse dorsal skin blood flow in this model. This technique consistently detected increased dorsal skin blood flow in Aldara, but not Vaseline-treated groups (Fig. 1*B*). Moreover, Aldara treatment mediated spontaneous behaviors in mice, identified as hind paw scratching, biting/licking of the treated dorsal area, and flinching of the dorsal area (Fig. 1*D–G*), indicative of nocifensive behaviors. The trends in hind paw scratching were inconsistent and variable throughout the 4 d of treatment (Fig. 1*E*). In contrast, biting/licking and flinching were found to be increased, in keeping with the skin pathologic changes as the days of exposure to Aldara progressed. This suggests that those mice experienced sensory discomfort, similar to the clinical condition (3). Hence, the role for nociceptors in this model was investigated further using RTX-mediated denervation to deplete the small-diameter nociceptors, important in pain and itch.

RTX-mediated sensory denervation resulted in successful sensory denervation maintained over the 4 d of skin treatment, as tested daily by checking for hind paw sensitivity to heat (Supplemental Fig. S1) (22). Despite evidence for the expression of TRPV1 in nonneuronal cells, including keratinocytes and immune cells (34, 35), RTX denervation reduced the expression of sensory markers, including TRPV1, TRPA1, SP, and α-CGRP mRNA expression in the DRGs, with no effect on their expression in the skin (**Table 1**). Additionally, the expression of TRPM8, TLR7, and TLR3 in the DRGs were not altered by RTX treatment (Table 1). Hence, RTX used in this study seemed to specifically deplete for TRPV1^+^ nociceptors. Sensory depletion led to significantly reduced skin thickness, acanthosis, and dorsal skin blood flow (Fig. 1*A–C*), consistent with the observed improvement in skin pathology macroscopically. We also observed an increase in IL-1β mRNA after 4 d with Aldara treatment, which was not affected by RTX treatment (Table 1). Additionally, IL-6 and IL-8 mRNA showed trends toward increased expression with Aldara treatment and was reduced in Aldara-treated skin with sensory denervation, whereas CCL5, an important chemokine in leukocyte recruitment, was decreased with RTX treatment (Table 1). We were unable to detect IL-23p19 and IL-17A mRNA expression and did not observe significant changes in TNF-α mRNA (Table 1), possibly because of the timing of the sample collection. Overall, our data confirmed the dual importance of those RTX-sensitive nociceptors in regulating both the inflammatory and pruritic aspects of this model.

**TABLE 1:** A summary of the mRNA expression of sensory mediators and inflammatory cytokines in the DRG and the skin

Sensory mediator	Vaseline (copies/μl)		Aldara (copies/μl)	
	Vehicle	RTX	Vehicle	RTX
DRG				
TRPV1	1899.9 ± 663.8	305.5 ± 126.4^###^	1536.2 ± 664.6	173.2 ± 51.1^###^
TRPA1	182.6 ± 58.1	91.9 ± 25.6^###^	170.1 ± 56.0	71.1 ± 15.2^###^
SP	8564.8 ± 2057.3	1870.7 ± 557.2^###^	6366.5 ± 4347.1	1771.3 ± 754.7^###^
α-CGRP	5070.9 ± 988.1	2375.8 ± 886.3^###^	1579.7 ± 442.2***	1364.6 ± 521.2***
TRPM8	257.1 ± 139.2	231.3 ± 84.5	276.6 ± 152.2	202.4 ± 69.9
TLR7	94.2 ± 62.7	118.0 ± 79.7	23.4 ± 44.1	47.5 ± 67.5
TLR3	66.3 ± 36.6	145.8 ± 125.5	129.9 ± 92.0	111.9 ± 86.2
Skin				
TRPV1	11.5 ± 13.8	8.1 ± 10.6	13.4 ± 12.8	38.1 ± 30.0
TRPA1	1.2 ± 0.9	1.2 ± 1.5	0.7 ± 0.5	1.3 ± 1.2
SP	26.2 ± 31.7	26.6 ± 21.1	4.9 ± 7.1	10.7 ± 9.3
α-CGRP	48.7 ± 15.8	70.5 ± 45.2	30.4 ± 9.5	40.1 ± 18.8
TRPM8	6.1 ± 8.5	5.8 ± 4.8	2.5 ± 5.8	3.7 ± 4.4
IL-1β	359.2 ± 146.3	431.4 ± 340.2	1266.8 ± 619.2	1758 ± 812.4
IL-8	119.4 ± 26.5	89.11 ± 22.9	154.7 ± 34.1	69.3 ± 18.0^###^
IL-6	359.3 ± 73.5	392.2 ± 109.0	453.7 ± 159.8	401.7 ± 100.3
TNF-α	145.0 ± 37.1	162.2 ± 71.6	123.7 ± 55.4	149.3 ± 67.9
CCL5	549.9 ± 289.9	332.3 ± 224.0	515.8 ± 378.7	62.9 ± 48.1**^,^^#^
IL-17A	Too few for analysis	Too few for analysis	Too few for analysis	Too few for analysis
IL-23	Too few for analysis	Too few for analysis	Too few for analysis	Too few for analysis

Data represent means ± sd and were analyzed by 2-way ANOVA with Bonferroni’s *post hoc* test. *n* = 5–7 animals/group. **P < 0.01, ***P < 0.001 Vaseline *vs.* Aldara, ^#^*P* < 0.05, ^###^*P* < 0.001 vehicle *vs.* RTX.

### Macrophages, but not neutrophils, act downstream of nociceptor involvement

**Figure 2:**
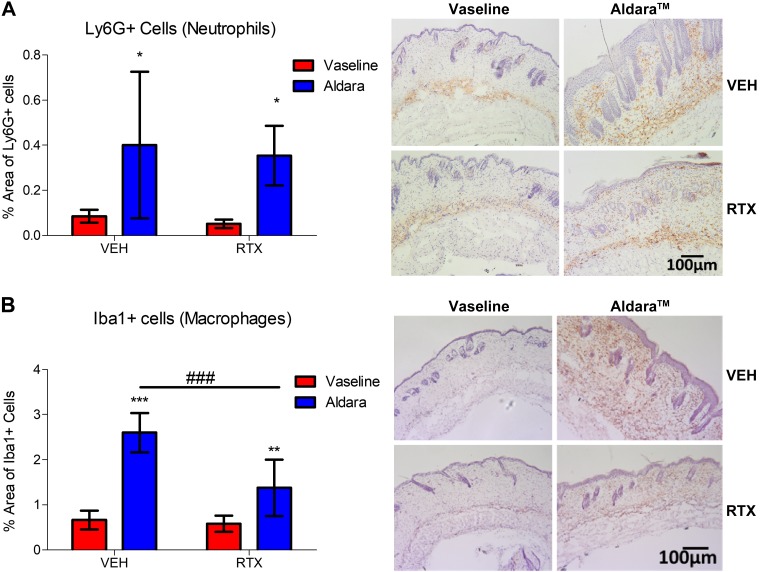
Sensory denervation protected against dermal macrophage (Iba1^+^ cells), but not neutrophil (Ly6G^+^ cells), influx. *A*) Grouped data of percentage of the area of dermal Ly6G^+^ cells and representative images of skin sections (right). Original magnification, ×10. Scale bar, 100 μm. Hematoxylin counterstaining. *B*) Grouped data of percentage of the area of dermal Iba1^+^ cells and representative images of skin sections (right). Original magnification, ×10. Scale bars, 100 μm. Hematoxylin counterstaining. Veh, vehicle. Graphs represent means ± sd, and data were analyzed by 2-way ANOVA with Bonferroni’s *post hoc* test. *n* = 5–7 animals/group.*P < 0.05, **P < 0.01, ***P < 0.001 between Vaseline *vs.* Aldara group, ^###^*P* < 0.001 between Veh *vs.* RTX groups.

**Figure 3:**
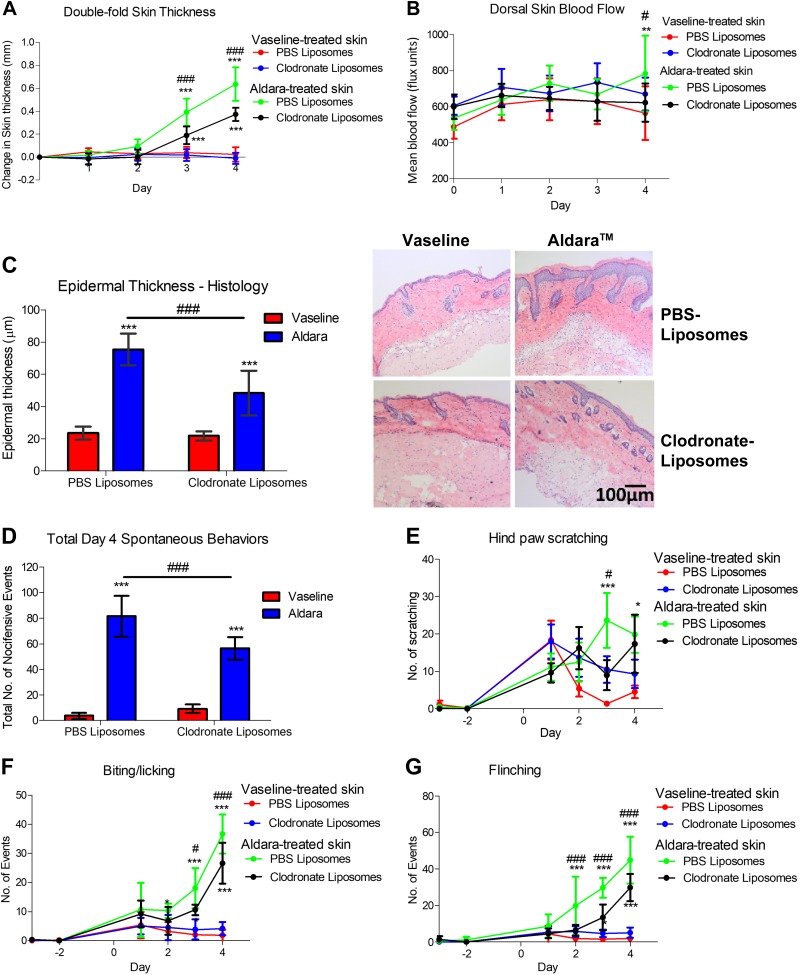
Macrophage depletion protected against psoriasis-like skin pathology and associated cutaneous discomfort. *A*, *B*) *In vivo* change in double-fold skin thickness (mm) (*A*) and dorsal skin blood flow (*B*). *C*) Epidermal thickness measured and representative images of H&E-stained skin samples sectioned at 10 μm thickness (right). Original magnification, ×10. Scale bar, 100 μm. *D*) Total spontaneous behaviors on d 4. *E*) Total number of hind paw scratching events. *F*) Total number of biting/licking events. *G*) Total number of flinching events. Graphs represent means ± sd, and data were analyzed by repeated-measures 2-way ANOVA with Bonferroni’s *post hoc* test; *n* = 5–6 animals/group. *P < 0.05, **P < 0.01, ***P < 0.001 between Vaseline *vs.* Aldara group, ^#^*P* < 0.05, ^###^*P* < 0.001 between Clodronate- *vs.* PBS-containing liposomes.

**Figure 4:**
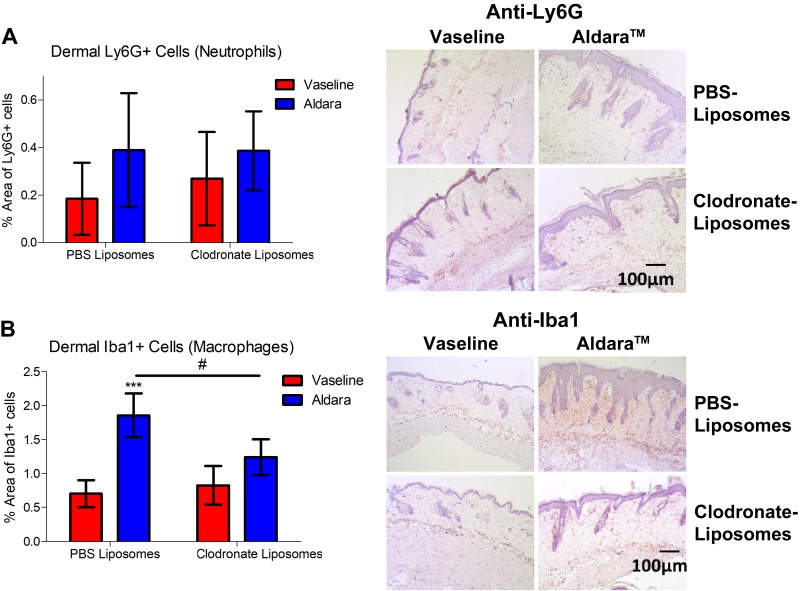
Macrophage depletion reduced dermal macrophage (Iba1^+^ cells), but not neutrophil (Ly6G^+^ cells) influx. *A*) Grouped data of percentage of area of dermal Ly6G^+^ cells and representative images of skin sections (right). Original magnification, ×10. Scale bar, 100 μm. Hematoxylin counterstaining. *B*) Grouped data of percentage of the area of dermal Iba1^+^ cells using automated analysis and representative images of skin sections (right). Original magnification, ×10. Scale bar, 100 μm. Hematoxylin counterstaining. Graphs represent means ± sd, and data were analyzed by 2-way ANOVA with Bonferroni’s *post hoc* test; *n* = 4–6 animals/group. ***P < 0.001 between Vaseline *vs.* Aldara group, ^#^*P* < 0.05 between clodronate- and PBS-containing liposomes group.

Because the mRNA expression data hinted toward a potential role for sensory nerves in regulating leukocyte recruitment, we sought to further elucidate the immunologic consequences underlying the observed protection. The skin sections were stained for inflammatory cell markers, focusing on neutrophils and macrophages. Ly6G^+^ neutrophils showed a variable, albeit significant increase, in Aldara-treated skin. Similarly, dermal Iba1^+^ macrophages were significantly increased in the Aldara-treated group (**Fig. 2*A*, *B***). Sensory denervation significantly reduced dermal macrophage, but not neutrophil, influx (Fig. 2). Hence, we sought to determine the role for dermal macrophage influx in driving Aldara-mediated skin inflammation downstream of nociceptor activation. Mice pretreated with clodronate liposomes, which selectively deplete macrophages (36), showed a significant improvement in skin pathology and erythema (**Fig. 3*A–C***). Immunohistochemical analysis confirmed that dermal macrophage influx was inhibited in the Aldara group without affecting dermal neutrophil expression, confirming the specificity of this depletion technique for macrophages (**Fig. 4**). Macrophage depletion significantly reduced the occurrence of the spontaneous behaviors (Fig. 3*D*), mainly for biting/licking events and flinching, with minimal effect on hind paw scratching (Fig. 3*E–G*). Hence, dermal macrophages have important roles downstream of nociceptor regulation of Aldara-induced skin inflammation, highlighting an interesting interaction between nociceptors and macrophages in both skin inflammation and associated cutaneous discomfort.

### Peroxynitrite-mediated protein nitrosylation has a significant role downstream of nociceptor regulation and macrophage-driven skin inflammation

**Figure 5:**
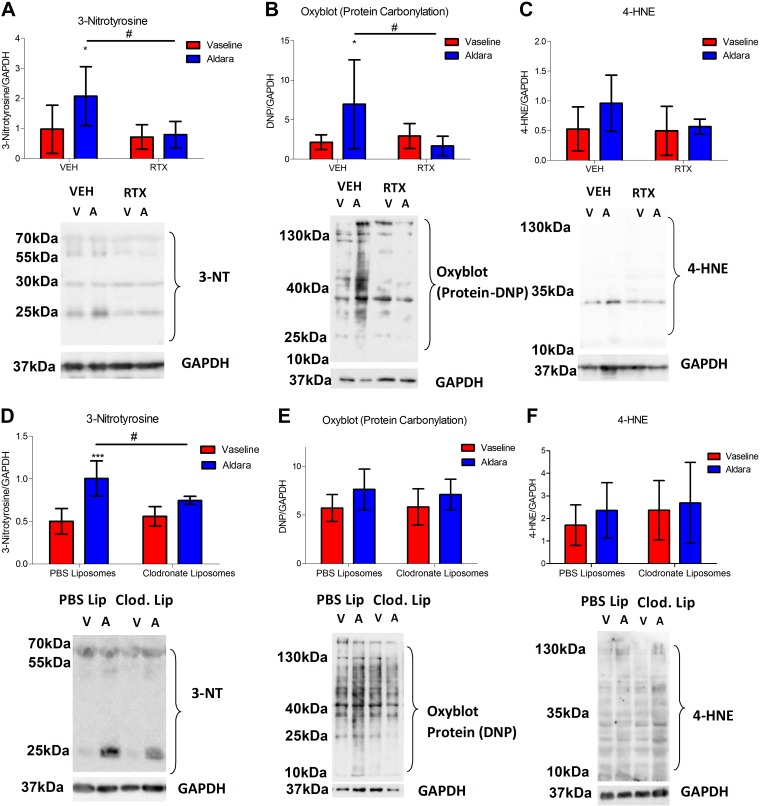
Sensory denervation and macrophage depletion protected against peroxynitrite-mediated oxidative stress in Aldara-induced skin inflammation. *A*, *D*) Grouped data for 3-NT expression and representative blot for 3-NT and GAPDH (bottom). *B*, *E*) Grouped data for protein carbonylation and representative blot for protein-dinitrophenylhydrazone (DNP) and GAPDH. *C*, *F*) Grouped data for 4‑HNE–protein adducts and representative blot for 4-HNE–protein and GAPDH (bottom). VEH, vehicle. Graphs represent means ± sd, and data were analyzed by 2-way ANOVA with Bonferroni’s *post hoc* test; *n* = 4–6 animals/group. *P < 0.05, ***P < 0.001 between Vaseline *vs.* Aldara group, ^#^*P* < 0.05 between VEH *vs.* RTX.

The downstream mediators associated with the inflammatory changes observed in the skin of this psoriasis model were further investigated. Clinically, psoriasis has been associated with increased oxidative stress markers, although their source is poorly understood (37). Hence, we first investigated whether cutaneous oxidative/nitrosative stress markers were altered in this model of psoriasis; 3-NT, a marker of peroxynitrite-dependent protein nitrosylation, was significantly increased in Aldara-treated skin (**Fig. 5*A*, *D***). Similarly, a second marker of oxidative stress, as indicated by protein carbonylation measured using the Oxyblot technique, was increased in lesional skin, albeit with higher variability (Fig. 5*B*, *E*). However, 4-HNE, a marker for lipid peroxidation, which may contribute to oxidative protein changes, was not significantly altered in the diseased skin (Fig. 5*C*, *F*), suggesting a minimal contribution for 4-HNE in this model.

Sensory denervation resulted in significantly reduced protein nitrosylation (Fig. 5*A*) and carbonylation (Fig. 5*B*), with minimal effect on 4-HNE markers (Fig. 5*C*). Because macrophages have been previously shown to have a significant role in the production of ROS/RNS under inflammatory settings (38–40), we sought to determine their effects on those oxidative/nitrosative markers in the skin. The protein nitrosylation marker (3-NT) was significantly decreased in response to the clodronate liposome treatment (Fig. 5*D*), whereas no significant difference was observed in protein carbonylation and 4-HNE expression (Fig. 5*E*, *F*). This suggests that macrophages partially contribute to the release of ROS/RNS and subsequent oxidative/nitrosative stress changes in the skin, involving peroxynitrite production.

### ROS/RNS directly contributes to skin pathology and skin inflammation

**Figure 6:**
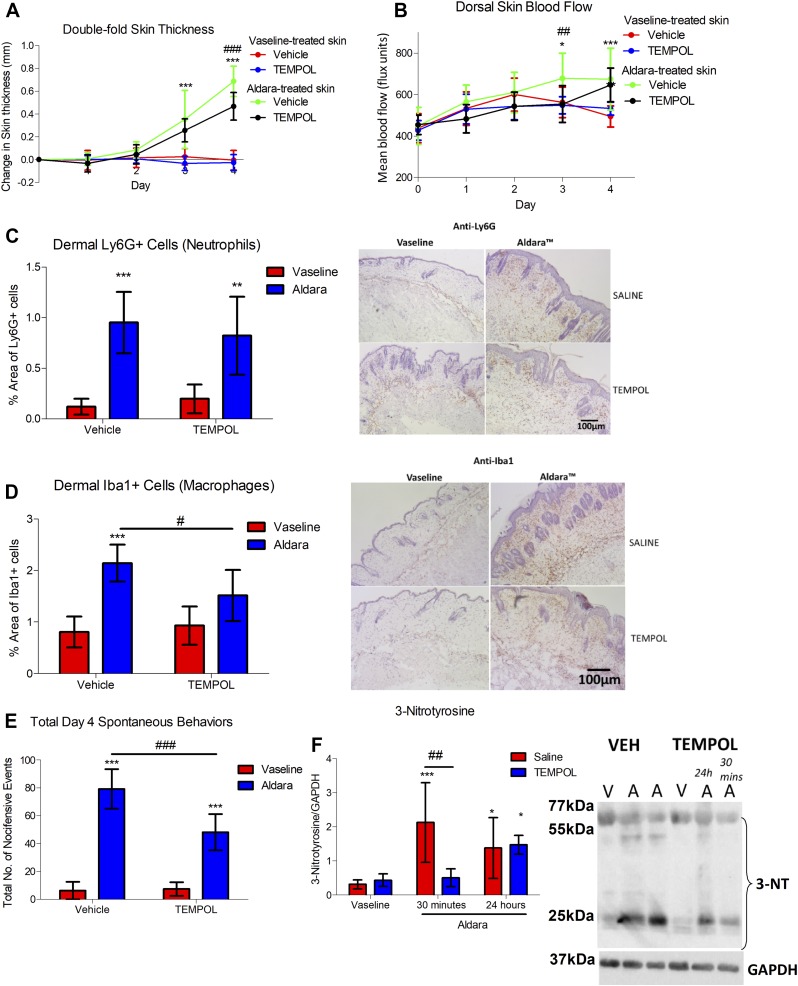
Tempol protected against Aldara-mediated skin pathology. *A*) Changes in double-fold skin thickness (mm). *B*) Dorsal skin mean blood flow (flux units). *C*) Grouped data of percentage of area of dermal Ly6G^+^ cells using automated analysis and representative images of skin sections (right). Hematoxylin counterstaining. *D*) Grouped data of percentage of area of dermal Iba1^+^ cells and representative images of skin sections (right). Hematoxylin counterstaining. *E*) Total d 4 spontaneous behaviors. *F*) Grouped data for 3-NT and representative blot for 3-NT and GAPDH (right). Graphs represent means ± sd and were analyzed by 2-way or repeated measures ANOVA with Bonferroni’s *post hoc* test; *n* = 4–7 animals/group. Original maginification, ×10. Scale bars, 100 μm. *P < 0.05, **P < 0.01, ***P < 0.001 between Vaseline *vs.* Aldara groups, ^#^*P* < 0.05, ^##^*P* < 0.01, ^###^*P* < 0.001 between VEH *vs.* tempol. A, Aldara, V, Vaseline.

Despite the reduction in oxidative/nitrosative stress in response to sensory denervation and macrophage depletion, the direct roles for oxidative/nitrosative stress in mediating skin pathology were unclear. Hence, to investigate that, tempol, a permeable SOD mimetic and antioxidant, was administered once daily before Aldara treatment to determine whether ROS/RNS directly contributed to the observed skin pathology and underlying inflammation. Repeated tempol treatment had a slight, but significant, effect on reducing skin thickness (**Fig. 6*A*** and Supplemental Fig. S2*A*) and dorsal skin blood flow during d 2–3 (Fig. 6*B*). In addition, the influx of dermal macrophages, but not neutrophils, was reduced with tempol treatment (Fig. 6*C*, *D*). Tempol also significantly reduced all aspects of the spontaneous behaviors associated with this model (Fig. 6*E* and Supplemental Fig. S2*B–D*), highlighting the importance of those markers in mediating both the skin inflammation as well as the sensory symptoms in this model. Immunoblotting in the skin of the tempol-treated group showed an inhibition of peroxynitrite-mediated protein nitrosylation within 30 min of treatment, which reversed back to the level in the diseased state at 24 h after tempol injection (Fig. 6*F*). Taking into account the lack of detected effects of tempol on carbonylation (Supplemental Fig. S2*E*), together with significant decreases in nitrosylation, this indicates that the protection by tempol on skin pathology was mediated by peroxynitrite-mediated nitrosylation. These findings suggest positive feedback interactions among sensory nerves, macrophages, and associated peroxynitrite production in skin inflammation, maintaining the proinflammatory state seen in this model of psoriasis.

### Neuropeptides are downstream of TRPA1 activation in the observed spontaneous behaviors in the Aldara model of psoriasis

To investigate the downstream sensory mechanisms and mediators, we used a dual genetic and pharmacological approach. The sensory markers chosen were those previously characterized by ourselves and others in inflammation and itch (13, 15, 41). Firstly, the mRNA expression of the sensory mediators was determined in the DRGs, which are the cell bodies of the cutaneous sensory nerve projection, as well in the treated dorsal skin by quantitative RT-PCR. That analysis was performed in samples from either control or sensory nerve-depleted (RTX-treated) mice that were treated with Aldara or vehicle. The expression of TRPV1, TRPA1, SP, and α-CGRP was significantly reduced with RTX denervation in the DRG, without any significant change in TRPM8 and TLR7 expression (Table 1). In contrast, the expressions of those markers were at a much lower level in the skin, and there was no significant change with either Aldara or RTX treatment. This further highlights the specificity of RTX in targeting a subset of TRPV1^+^ TRPA1^+^ neuropeptide-positive sensory nerves with minimal effects in nonneuronal cells (Table 1).

**Figure 7:**
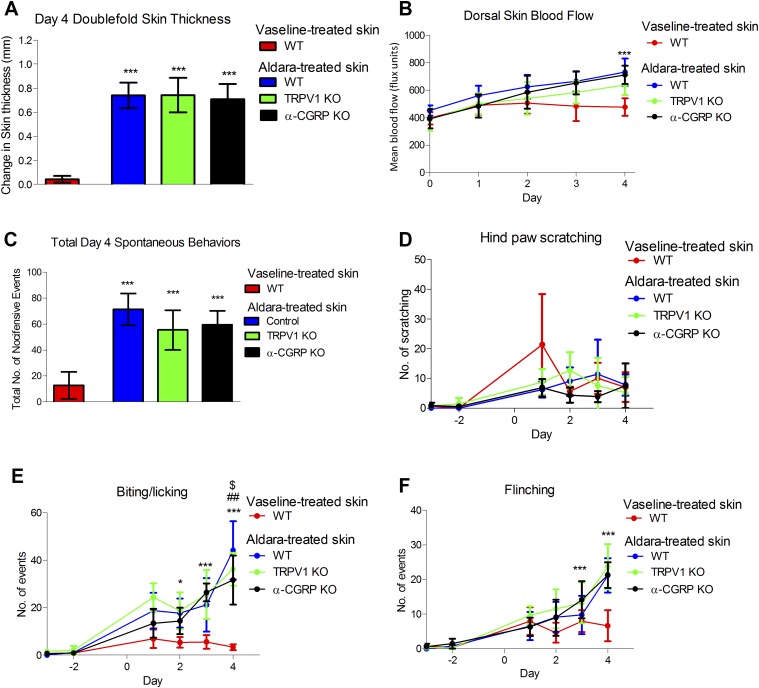
TRPV1 and α-CGRP deletion showed a significant reduction in spontaneous behaviors independent of the development of skin pathology. *A*) Change in double-fold skin thickness (mm). *B*) Dorsal skin blood flow. *C*) Total d 4 spontaneous behaviors in TRPV1 wild type (WT), TRPV1 KO, or α-CGRP KO groups. *D*) Total number of hind paw scratching events. *E*) Total number of biting/licking events. *F*) Total number of flinching events. Graphs represent means ± sd, and data were analyzed by 1-, 2-way, or repeated-measures ANOVA with Bonferroni’s *post hoc* test; *n* = 5–6 animals/group. **P < 0.01, ***P < 0.001 Vaseline *vs.* Aldara, ^##^*P* < 0.001 WT *vs.* α-CGRP KO, ^$^*P* < 0.05 WT *vs.* TRPV1 KO.

Hence, we further investigated the contributions of TRPV1, SP, α-CGRP, and TRPA1 in the observed skin pathology and spontaneous behaviors. Here, the deletion of TRPV1 or α-CGRP gene failed to inhibit the development of skin pathology in the Aldara model (**Fig. 7*A*, *B***), with only minimal changes in the total d 4 spontaneous behaviors in the TRPV1 KO mice (Fig. 7*C*) and a small, but significant, reduction in biting/licking events, with no effect on the other behaviors (Fig. 7*D–F*). Similarly, α-CGRP KO mice also showed a slight reduction in biting/licking events with minimal effects on scratching and flinching (Fig. 7*D–F*), which may indicate minimal involvement of the TRPV1-α-CGRP pathways.

**Figure 8:**
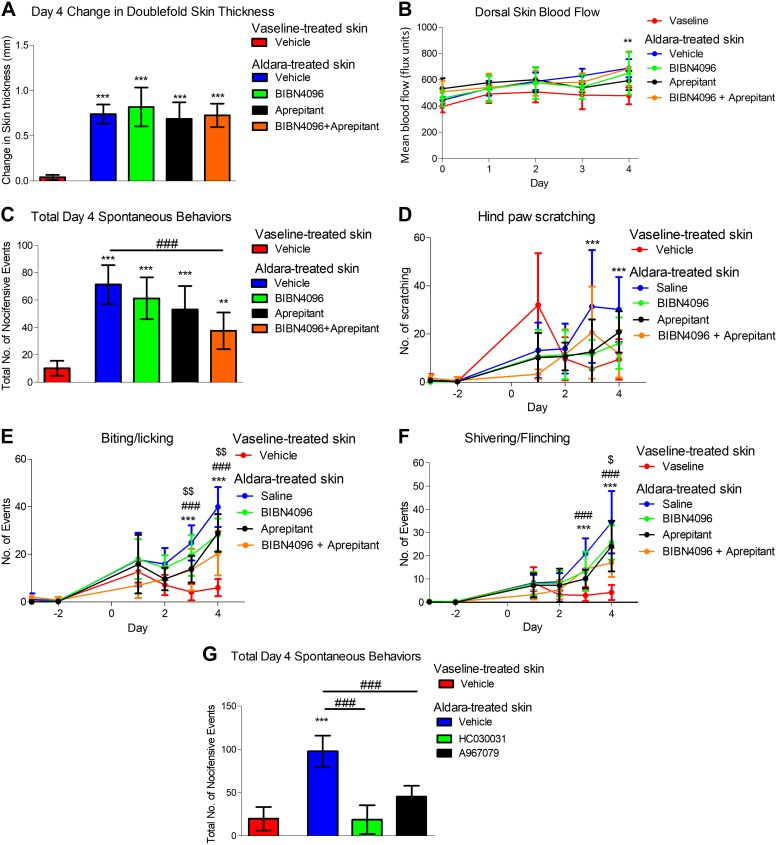
Neuropeptides SP/CGRP and TRPA1 contribute to cutaneous discomfort, independent of the development of skin pathology. *A*) Change in double-fold skin thickness (mm). *B*) Dorsal skin blood flow. *C*) Total d 4 spontaneous behaviors in response to various treatment regime. *D*) Total number of hind paw scratching events. *E*) Total number of biting/licking events. *F*) Total number of flinching events. *G*) Total d 4 spontaneous behaviors in response to acute TRPA1 antagonist administration. Graphs represent means ± sd, and data were analyzed by 1-way or repeated-measures ANOVA with Bonferroni’s *post hoc* test; *n* = 5–7 animals/group. **P < 0.01, ***P < 0.001 Vaseline *vs.* Aldara, ^###^*P* < 0.001 vehicle *vs.* treatments (HC030031 or the different combinations of BIBN4096 and aprepitant), ^$^*P* < 0.05, ^$$^*P* < 0.01 between BIBN4096+ aprepitant *vs.* aprepitant.

Using pharmacological approaches, we further elucidated the roles for neuropeptides SP and CGRP in this model using the clinically available NK1 receptor antagonist (Aprepitant) and CGRP receptor antagonist (BIBN4096). Despite minimal effects on skin pathology (**Fig. 8*A, B***), we showed a reduction in biting/licking and flinching in response to separate Aprepitant treatment (Fig 8*C*–*F*). A decreasing trend, which was not significant, was also observed with BIBN4096 (a CGRP receptor antagonist targeting α- and β-CGRP) treatment alone (Fig. 8*D–F*). Coadministration of both CGRP and SP antagonists has the greatest antipruritogenic efficacy (Fig. 8*C*). Interestingly, the beneficial effects of those treatments seemed independent of the skin pathology, as all of the tested agents failed to modify the skin pathology (Fig. 8*A*, *B*). Hence, this suggests that the release of neuropeptides is important in mediating psoriasis-associated spontaneous behaviors and cutaneous discomfort. To determine the mechanisms triggering neuropeptide release, the role for TRPA1, which is expressed in ∼60–70% of TRPV1^+^ neuropeptide-positive nociceptors and known to mediate itching (16) was investigated. Both TRPA1 antagonists (HC030031 and A967079) significantly inhibited spontaneous behaviors (Fig. 8*G*) when administered immediately (within 30 min) before assessment, suggesting an important role for TRPA1 in mediating cutaneous discomfort in the Aldara model of psoriasis.

## DISCUSSION

This study shows an important role for TRPV1^+^TRPA1^+^SP/CGRP^+^ sensory nerves in mediating both skin pathology and associated cutaneous discomfort in the Aldara model of psoriasis, consistent with previous clinical evidence for the potential of sensory nerves to influence skin lesions and consequential cutaneous discomfort complications in this disease (3, 42). Herein, we identify a key dermal macrophage–ROS/RNS component that is critical to the ongoing pathology, with evidence that peroxynitrite and subsequent production of oxidative/nitrosative stress acts to amplify the contribution of sensory nerves to the inflammation. This, in turn, initiates TRP- mediated activation of sensory nerves to release SP and CGRP, which contribute to the sensory discomfort associated with psoriasis. The Aldara model used in the present study reflected a later inflammation phase, as indicated by the consistent and significant increase in dermal macrophages, with a slight and variable increase in dermal neutrophils (43). We observed a significant increase in IL-1β, and trends of increased IL-6 and IL-8 mRNA in the Aldara-treated skin, although we did not detect significant changes in IL-23p19, TNF-α, and IL-17A expression, in contrast to previous findings (6, 19, 24). A possible explanation for this may be due to the time point during which the skin samples were collected. Van der Fits *et al.* (6) showed some cytokines to peak at earlier time points, which may have been missed by collecting the skin samples on d 4. Our findings suggest that sensory denervation is protective during the later phase of skin inflammation, expanding on the discovery of Riol-Blanco *et al.* (19) that a regulatory role for nociceptors during the early skin inflammation phase involving dermal dendritic cells and γδ T cells. In keeping with previous studies, we show that macrophages have an essential role in psoriasis-like skin inflammation, consistent in various murine models of psoriasis (43–47). Our discovery that RTX-mediated sensory nerve depletion reduced the expression of various inflammatory mediators associated with leukocyte recruitment and subsequently inhibited the dermal macrophage influx provides evidence for one of the mechanisms by which sensory nerves mediate this skin pathology.

To further understand the sensory nerve-dermal macrophage inflammatory link in the Aldara model, we investigated the contribution of ROS/RNS, an expected consequence of macrophage influx under inflammatory conditions (38, 39). The lesional skin samples showed an increase in oxidative/nitrosative stress, without any significant changes observed in 4-HNE expression, consistent with the knowledge that oxidative stress markers are increased in both murine models and clinical psoriasis (48, 49). Our results showed that sensory denervation or macrophage depletion with clodronate liposomes reduced 3-NT formation in Aldara-treated skin, consistent with the implication that peroxynitrite is one of the major mediators produced downstream because of sensory nerve-mediated activation of macrophages. However, the contribution of other cell types, such as neutrophils and keratinocytes, in the production of ROS/RNS, must also be considered (50, 51). The influence of the macrophage/ROS pathway on the pathophysiology observed in this model was further confirmed *in vivo* by administering tempol, a cell-permeable SOD mimetic, which resulted in a slight improvement in skin pathology as well as reduced dermal macrophage recruitment (Fig. 6). Despite prior evidence showing the benefits of antioxidant treatments in acute inflammatory and pain models (52, 53), we now demonstrate, with the biomarker 3-NT, that peroxynitrite directly contributes to dermal macrophage influx and skin pathology *in vivo* to support prior *in vitro* findings (54, 55). Indeed, macrophage depletion and antioxidant treatment only reduced, but did not ablate, the spontaneous behaviors. This is perhaps unsurprising because psoriasis is a complex inflammatory condition. However, we are the first, to our knowledge, to show a link between macrophages and spontaneous behaviors in a skin inflammatory model. Currently, we have not further elucidated the mechanisms by which sensory nerves regulate macrophage influx into the skin. However, a recent study highlighted the importance of microRNA in mediating sensory nerve-macrophage communication in a model of nerve injury (56), although their roles in other types of conditions, such as psoriasis, remain to be determined.

Additionally, the sensory discomfort associated with psoriasis is an established debilitating component, with antihistamines and anti-inflammatory treatments having limited effects. A recent meta-analysis highlighted a major step forward in taking into account the measurement of sensory discomfort as one of the main outcomes to be measured during the development of novel antipsoriatic treatments (2, 5). Here, we performed a detailed analysis of the spontaneous behaviors that are considered to be in keeping with cutaneous discomfort and nocifensive behaviors in the mouse. Aldara-induced psoriasis triggered scratching behaviors and was first reported while this study was in progress. Sakai *et al.* (7) showed that a 7-d treatment with Aldara on the nape of the neck of C57/BL6J mice triggered scratching and alloknesis, indicative of the need to scratch. By comparison, we observed variable, inconsistent hind paw scratching when Aldara was applied on the mid-to-low area of the dorsal skin, but biting/licking events and flinching behaviors were observed, indicative of sensory discomfort (3, 28). The different observations may be due to the different sites of the lesions as it has been shown that the injection of pruritogenic stimuli, such as serotonin, into the hind paws resulted in an itch-associated biting response (57) because of the inaccessibility to hind paw scratching of the affected area (57). In addition, clinical psoriasis is often associated with cutaneous pain and discomfort, which is often described as biting and tingling, unlike the pruritus associated with atopic dermatitis (3). Hence, we propose that this model of psoriasis mimics the cutaneous discomfort observed in humans.

We confirmed the expected involvement of the sensory nerves as RTX-mediated denervation inhibited the occurrence of the spontaneous behaviors. Additionally, both macrophage depletion and tempol significantly reduced that response. Tempol appeared to have a larger inhibitory effect on cutaneous discomfort than on skin pathology and inflammation, suggesting direct effects of ROS/RNS in mediating the spontaneous behaviors independent of the development of skin pathology, consistent with previous findings that showed macrophage-mediated ROS/RNS production to have major roles in chemotherapy-induced pain (58) and in chronic orofacial pain (40). We are the first, to our knowledge, to illustrate this interaction among sensory nerve activation, macrophages, and ROS/RNS in skin inflammation and associated cutaneous discomfort, highlighting novel therapeutic targets and mechanisms underlying the sensory symptoms in psoriasis.

Having established a link between sensory nerve depletion and the underlying inflammatory interactions and mediators, we sought to determine whether the sensory components, such as TRP channels and neuropeptides, contributed to regulating the inflammatory response. There were also no significant changes in the expression of TLR7 and TLR3, both previously shown to mediate pruritus (59, 60) in Aldara or sensory-denervated groups, suggesting that the reduction in spontaneous behaviors was unlikely to be due to the loss of the expression of those receptors in the DRG and possibly mediated by other sensory mediators. Previous studies have shown those mediators to be involved in skin and as part of the neurogenic inflammatory component in a range of nonpsoriasis models, but none have investigated this pathway in a model of psoriasis (21, 41, 61, 62). Despite a significant decrease in TRPV1 mRNA expression in the DRG, TRPV1 deletion failed to significantly inhibit the development of skin pathology and cutaneous discomfort in the Aldara model. Despite initial interest in the contribution of TRPV1 in itching, TRPV1 has been mainly associated with histamine-dependent itching (63), whereas a TRPV1 antagonist failed to inhibit histamine-dependent and -independent itching in humans (64). Hence, this suggests that TRPV1 does not have a major role in this model of psoriasis and the cutaneous discomfort. Similarly, despite previous evidence for the potential roles of neuropeptides SP and CGRP in human psoriatic skin (65, 66) and a transgenic murine model of psoriasis (11), we failed to detect a significant increase in neuropeptide mRNA expression in the Aldara-treated skin and DRG or an improvement in the skin pathology in the Aldara model. In fact, we found that only α-CGRP mRNA levels were reduced in the DRG in response to Aldara treatment, although that observation seemed specific for α-CGRP expression, as the other sensory markers remained similar among the skin treatment groups.

We are confident that the antagonists administered were used at effective concentrations because the spontaneous behaviors were substantially inhibited, most effectively when both neuropeptide antagonists were administered simultaneously, suggesting a contribution of both neuropeptides. Indeed, the increase in neuropeptides in psoriasis has been shown to be associated with expression in the cutaneous nerves (65, 67) and thought to indicate the severity of pruritus in patients with psoriasis (68), whereas acute coadministration of SP and CGRP resulted in an enhanced itch sensation in comparison to SP alone (69). Recently, targeting SP clinically has been shown to be an effective anti-pruritic treatment in various conditions, including skin cancer-associated itch (70) as well as in patients with various skin disorders such as atopic dermatitis and prurigo nodularis (71), although negligible evidence is currently available in psoriasis-associated discomfort. Hence, these findings may lead to an exciting novel indication for neuropeptide antagonists in psoriasis-associated sensory discomfort.

Lastly, we investigated whether TRPA1, established as a potential trigger for neuropeptide release in tissues that include mouse skin (61, 72), was involved in mediating the spontaneous behaviors in this psoriasis model. We chose to investigate the role for TRPA1 as a potential sensory-activating mechanisms upstream of neuropeptide release in our model because of its activation by various ROS/RNS, including peroxynitrite, in skin (61, 73). In addition, acute administration of TRPA1 antagonists (within 30 min to 5 h after treatment) significantly inhibited inflammatory-mediated itch (41) as well as in murine models of contact dermatitis (15). Our study highlights an important acute role for TRPA1 in mediating psoriasis-associated spontaneous behaviors in the Aldara model because treatments with 2 structurally distinct TRPA1 antagonists significantly inhibited the observed spontaneous behaviors in this model, highlighting the importance of TRPA1 in psoriasis-associated sensory discomfort. This study has highlighted an important acute contribution of the TRPA1-sensory nerve pathway in psoriatic sensory discomfort mediated by neuropeptide-dependent mechanisms. It is important to note, however, that we have recently shown that TRPA1 deletion or the long-term administration of a TRPA1 antagonist throughout the induction and maintenance phases of the Aldara model were associated with a worsened skin phenotype, with evidence suggesting that the phenotype was nonneuronal TRPA1, such as those found in immune cells (74, 75). This highlights the complex regulatory role of TRPA1 in skin inflammatory conditions (20). The present findings build on previous findings to potentially suggest involvement of TRPA1 at multiple sites (such as neuronal and nonneuronal) in this model of psoriasis. With this, the combined results of Kemeny *et al.* (20) and the present study indicate that neuropeptide antagonists may be a beneficial approach to treating cutaneous discomfort in patients with psoriasis. Imiquimod may also act as a TRPA1 agonist (20, 76), but in the present study, we found that other potential TRPA1 agonists (such as oxidative stress products) are involved because their inhibition was observed to reduce cutaneous discomfort.

To summarize, we propose that Aldara-mediated psoriasis triggers sensory nerve-mediated dermal macrophage influx and subsequent production of protein nitrosylation. This contributes to further dermal macrophage influx, resulting in positive feedback mechanisms and the maintenance of the inflammatory phenotype. Additionally, the production of ROS/RNS, with evidence here for peroxynitrite, led us to suggest that this triggers the activation of TRPA1. This initiates the release of neuropeptides SP and CGRP from the sensory terminals to mediate psoriasis-associated cutaneous discomfort. Hence, we highlight the complex nature of events in the Aldara model of psoriasis and the significant interactions between sensory nerves and macrophages in mediating psoriasis-like skin inflammation in a ROS/RNS-dependent manner, which further contributes to the sensory discomfort in psoriasis, highlighting their potential as novel therapeutic targets for this condition.

## Supplementary Material

This article includes supplemental data. Please visit *http://www.fasebj.org* to obtain this information.

## Abbreviations

3-NT: 3-nitrotyrosine
4-HNE: 4-hydroxynonenal
CCL5: C-C motif chemokine ligand 5
CGRP: calcitonin gene-related peptide
DAB: 3,3′-diaminobenzidine
DRG: dorsal root ganglion
GAPDH: glyceraldehyde 3-phosphate dehydrogenase
H&E: hematoxylin and eosin
Iba1: ionized calcium-binding adapter molecule 1
KO: knockout
Ly6G: lymphocyte antigen 6 complex locus G6D
RNS: reactive nitrogen species
ROS: reactive oxygen species
RTX: resiniferatoxin
SOD: superoxide dismutase
SP: substance P
tempol: 4-hydroxy-2,2,6,6-tetramethylpiperidine-*N*-oxyl
TRPA1: transient receptor potential ankyrin 1
TRPM8: transient receptor potential melastatin 8
TRPV1: transient receptor potential vanilloid 1
