# Supplementary material for: Sensory nerves mediate spontaneous behaviors in addition to inflammation in a murine model of psoriasis
Source: FASEB J. 2018 Sep 11;33(2):1578–94. doi: 10.1096/fj.201800395RR (PMC6338626; doi:10.1096/fj.201800395RR)
Supplement: Supplementary file 1 [file fj.201800395RR.sf1.docx]

**Supplementary figures**

**Supplementary Figure 1.** **Hind paw withdrawal latency (s) to heat (55^o^C) was increased in RTX-treated groups.** Paw withdrawal latency (s) was defined as the time taken to show pruritic behaviors, including hind paw licking, flinching, or jumping within a 30-second observation timeframe. If no response was observed within 30 seconds, mice were removed and withdrawal latency was recorded as 30 seconds. Graph represents mean ± SD and data were analyzed by repeated measures 2-way ANOVA with Bonferonni’s *post hoc* test. n= 6-7 per group, *** p<0.001 compared to day 0 latency.


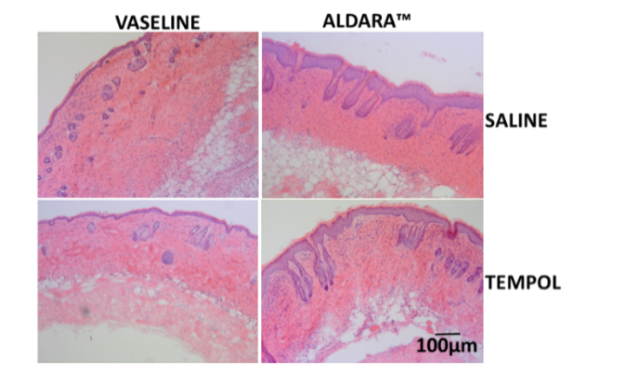


**A)**

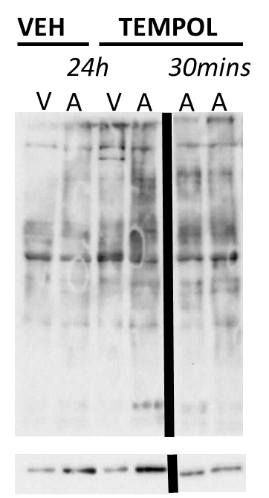


**GAPDH**

**D)**

**E)**

**Oxyblot (Protein-DNP)**

**B)**

**C)**

**Supplementary Figure 2. Effects of TEMPOL on skin thickness, spontaneous behaviors and protein carbonylation.** A) Grouped data for epidermal thickness (Inset: representative images of H&E-stained skin samples sectioned at 10μm thickness, 10X magnification). B) Total hind paw scratching. C) Total number of biting/licking (defined as bringing the mouth and nose towards the treated dorsal skin). D) Total number of flinching (defined as a rotating/flipping of the dorsal area). E) Grouped data for protein carbonylation and (inset) representative blot for Protein-DNP and GAPDH. Graph represents mean ± SD and data were analyzed by repeated measures or 2-way ANOVA with Bonferonni’s *post hoc* test. n= 5-7 per group, *** p<0.001 compared to Day 0 latency.
